# Trends and projections in adenocarcinoma and squamous cell carcinoma of the oesophagus in England from 1971 to 2037

**DOI:** 10.1038/s41416-018-0047-4

**Published:** 2018-03-22

**Authors:** Judith Offman, Francesca Pesola, Peter Sasieni

**Affiliations:** 1 Faculty of Life Sciences & Medicine, School of Cancer & Pharmaceutical Sciences, Innovation Hub, Guys Cancer Centre, Guys Hospital, King’s College London, Great Maze Pond, London, SE1 9RT UK; 2 Centre for Cancer Prevention, Wolfson Institute of Preventive Medicine, Barts and The London School of Medicine and Dentistry, Queen Mary University of London, Charterhouse Square, London, EC1M 6BQ UK

**Keywords:** Epidemiology, Cancer epidemiology, Oesophageal cancer

## Abstract

**Background:**

The aim of this study was to assess the incidence and trends of oesophageal adenocarcinomas (OACs) and squamous cell carcinomas (OSCCs) in England from 1971 to 2037.

**Methods:**

Data on 220,026 oesophageal cancers diagnosed in England between 1971 and 2013 were extracted. Multiple imputation was used to predict morphology data were missing. Incidence rates were modelled and extrapolated to 2037 using age–period-cohort models.

**Results:**

The OAC age-standardised incidence rate (ASRs) increase was greatest from 1972 to 1992 (from 4.8 to 12.3 for men and 1.1 to 3 per 100,000 for women) and slowed from 1992 to 2012 (with an increase to 17 for men and 3.8 per 100,000 for women). OSCCs rates decreased from 7.5 to 4.9 from 1972 to 2012 for men. For women, ASRs increased from 5.5 to 5.9 between 1972 and 1992 and then decreased to 4.7 per 100,000 until 2012. Rates until 2032 are predicted to stay stable for OACs and further decrease for OSCCs.

**Conclusions:**

Imputing missing morphology allowed accurate and up-to-date estimates of trends and projections. We observed a slowing down of the increase in OAC ASRs and an overall decrease in OSCC ASRs.

## Introduction

There has been a dramatic rise in the incidence of oesophageal cancer in the developed world over the last 30 years making it now the 8^th^ most common cancer worldwide.^1^ Furthermore, it has a dismal prognosis with age-standardised net survival being less than 50% at 1-year and less than 20% at 5-years.^2^ The vast majority of oesophageal cancers occur as either squamous cell carcinomas (OSCCs) or adenocarcinomas (OACs).^3^ In 2012, OSCCs occurred most commonly in South-Eastern and Central Asia, whereas the highest incidence of OACs were in high income countries, specifically Northern and Western Europe, North America, and Oceania.^1^ The highest incidence of OACs worldwide occurred in the UK. Furthermore, the incidence of OACs has increased dramatically in the last 30 years in some populations, whereas the incidence of OSCCs has decreased.^4–6^

The natural histories of OACs and OSCCs differ substantially. OSCCs usually arise from the stratified squamous epithelial lining in the upper two thirds of the oesophagus, and the main risk factors are smoking and alcohol.^7^ OACs, on the other hand, mainly develop in the lower third of the oesophagus originating from Barrett’s mucosa. Barrett’s oesophagus (BO), a complication of gastro-oesophageal reflux disease (GORD), predisposes patients to OAC, but the risk of malignant progression is low.^8,9^ In addition, other factors like obesity have been linked with OACs.^3,10^ Acid suppression using either proton pump inhibitors (PPIs) or H2 receptor antagonists (H2RAs) are the main treatments used in the management of GORD, and use of PPIs have been found to reduce the risk of malignant progression from Barrett’s to OAC.^11^

Classification of these different cancer types is based on morphology; however, morphology has frequently been missing from UK cancer registry records, especially for cancers diagnosed pre-1990, but also for later time periods.^12,13^ The aim of this study was to assess the incidence and trends of OACs and OSCCs in England from 1971 (when the earliest data are available) to 2013 using an age–period-cohort (APC) model after estimating missing morphology, and to estimate the future burden until 2037. Several groups have estimated trends and future projections of OAC and OSCC rates using APC models. However, they have not allowed for trends in cancers with unrecorded morphology. Here we use multiple imputation for cancers with missing morphology to more accurately estimate trends and projections. Furthermore, due to the linear dependence of age, period, and cohort in the APC model, it is not possible to identify the separate impact of age, period, and cohort effects. We explore the impact of simpler models as well as reallocation of the non-identifiable linear effect to better understand how these age, period, and cohort effects influenced previous and, therefore, will influence future OAC and OSCC trends. Understanding current trends and projecting future burden of both OAC and OSCC will play a key role in planning the delivery of cancer services. Specifically, as OACs and OSCCs have different risk factors, understanding the trends for these two subtypes separately will allow planning suitable cancer prevention interventions.

## Materials and methods

### Cancer registration and population data

Aggregate data on all available records of malignant cancers of the oesophagus diagnosed in England between 1971 and 2013 were provided by the Public Health England (PHE) Office of Data Release. Cancer data were provided by single year of diagnosis, sex and 5-year age-group. From 1971 to 1990 the cancer registries used the International Classification of Diseases Edition 9 (ICD-9) codes to record anatomical categories. From 1991 onwards the 10th Edition (ICD-10) has been used.^14^ The ICD-9 and ICD-10 codes for the anatomical sub-sites respectively are: cervical (150.0/C15.0), thoracic (150.1/C15.1), abdominal (150.2/C15.2), upper third (150.3/C15.3), middle third (150.4/C15.4), lower third (150.5/C15.5), overlapping lesion (150.8/C15.8) and unspecified (150.9/C15.9). Mid-year population estimates ^15^ and 2012-based population projections up to 2037 ^16^ for England were obtained from the Office for National Statistics.

For cancers diagnosed from 1971 to 1990 cancer morphology types were recorded based on the Manual Of Tumour Nomenclature and Coding (MOTNAC) ^17,18^ or the first edition of the International Classification of Diseases for Oncology (ICD-O-1).^19^ The second and third editions of the International Classification of Diseases for Oncology (ICD-O-2 and ICD-O-3) ^20,21^ have been used from 1991 onwards. Where ICD-O codes had been used morphology types were defined as follows; OSCCs: 8050–8078 and 8083–8084; OACs: 8140–8141, 8143–8145, 8190–8231; 8260–8263, 8310, 8410, 8480–8490, 8559–8551, 8570–8574, and 8576. For cancers with missing morphology information multiple imputation using a multinomial logistic regression model was used to predict the probability of morphology categories: OAC, OSCC or other (More details on multiple imputation methods can be found in the Supplementary Appendix Methods Section 1).

### Statistical analysis

Cancer incidence rates were modelled and extrapolated to 2037 fitting separate models for OACs and OSCCs by sex using the APC model as described previously.^22,23^ In brief, the basic APC model is:

*λ*(age, period) = *g*^−1^{*ƒ*_A_(age) + *ƒ*_P_(period) + *ƒ*_C_(cohort)}

where *λ* is the incidence rate as a function of age and calendar period, *g* is the link function and *ƒ*_A_, *ƒ*_p_ and *ƒ*_C_ are functions of age, period (i.e. year of incidence) and cohort (i.e. year of birth), respectively. Both logarithmic and ‘power-5’ (i.e. *g*^−1^(*x*)=*x*^5^ as used by Møller et al ^24^) functions were used as the link function *g*, as both had previously offered a good fit to site-specific cancer data in adult populations.^22^ Cubic splines were used for the functions *ƒ*_A_, *ƒ*_p_ and *ƒ*_C_ as they offer greater flexibility and more realistic projections under the assumption that changes occur gradually. Analyses were carried out in Stata 13 using a revised apcspline command, which allows fitting an age–drift model, which was not possible in the previous version.^23,25^ More details on the APC model and comparisons between age, age–drift (AD), age–period (AP), age–cohort (AC) and APC models are described in Section 2 of the Supplementary Appendix.

Incidence rates were standardised using the European standard population to calculate age-standardised rates (ASRs).^26^

## Results

There were 220,026 records of malignant cancers of the oesophagus diagnosed in England between 1971 and 2013. Of these, 87,650 had been recorded as OACs (67,505 in men; 20,145 in women) and 69,815 as OSCCs (31,853 in men; 37,962 in women). All other histological subtypes were rare with only 5020 cases (2621 in men and 2399 in women) making up about 2.3% of all oesophageal cancers. Morphology data were missing for 57,541 cases (32,333 in men and 25,208 in women). As ignoring cancers with missing morphology would seriously underestimate the true incidence of OACs and OSCCs, multiple imputation was used. Imputations took into account age, gender, year of diagnosis, cancer sub-site and basis of diagnosis—see Section 3 in the Supplementary Appendix for more details. The distribution of the three morphology subtypes was very similar when we included the imputed data compared to the raw data (see Supplementary Appendix Section 3 Figure S3). Comparisons of trends and projections with and without MI for both OACs and OSCCs can be found in Section 4 of the Supplementary Appendix. Cancers with imputed morphology were included in all of the following analysis.

The APC model provided a good fit for the data (see Section 5 in the Supplementary Appendix) and was used for our main analyses presented below. In addition, alternative models were explored in a sensitivity analysis.

### Oesophageal cancer trends from 1971 to 2013

**Fig. 1:**
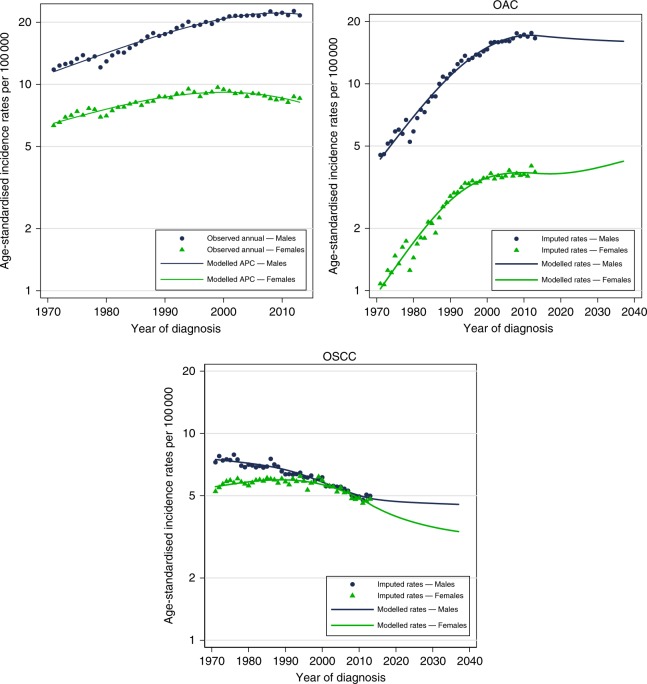
Incidence rates for all oesophageal cancers combined and OACs and OSCCs separately for men and women in England (log scale). **a** Annual and modelled incidence rates of all recorded malignant oesophageal cancers from 1971 to 2013. **b** Annual and modelled OAC incidence rates including cancers with estimated morphology for men and women from 1971 to 2013 and projected rates until 2037. **c** Annual and modelled OSCC incidence rates including cancers with estimated morphology for men and women from 1971 to 2013 and projected rates until 2037. Dots / triangles represent observed rates; lines represent modelled rates using the APC model. ASR age-standardised incidence rates, ESP European standard population, OAC adenocarcinoma, OSCC squamous cell carcinoma

ASRs for oesophageal cancer (all morphology types) for men have been increasing most significantly between the late 1970s and mid-2000s, and have started to level off now (Fig. 1a). ASRs for women have increased until the early 1990s when they stared to level off and then decreased again slightly since the early 2000s. However, when looking at OACs and OSCCs separately, opposing trends can be observed. Observed and modelled incidence rates for OACs and OSCCs by gender from 1971 to 2013 and projected rates until 2037 are presented in Fig. 1b, c. Table 1 shows the number of cases (including imputed cases) and ASRs for 1972, 1992 and 2012 as three-year averages for all ages combined for men and women.

**Table 1:** Age-standardised incidence rates and numbers of oesophageal adenocarcinomas and squamous cell carcinomas in men and women for three-year moving averages identified by mid-year

	**Average number of cases**							**Age-standardised incidence rate**						
	**Number**				**Total change**			**Rate per 100,000**				**Total change**		
	1972*	1992*	2012*	2032*	1972*–92*	1992*–2012*	2012*–2032*	1972*	1992*	2012*	2032*	1972*– 92*	1992*– 2012*	2012*–2032*
**Male**														
**OAC**	659	1981	3659	5191	201%	85%	42%	4.8	12.3	17	16.2	160%	38%	−5%
**OSCC**	971	1016	1058	1460	5%	4%	38%	7.5	6.4	4.9	4.6	−15%	−22%	−7%
**Female**														
**OAC**	221	706	1,002	1531	219%	42%	53%	1.1	3	3.8	4	168%	24%	6%
**OSCC**	1073	1338	1,237	1,338	25%	−8%	18%	5.5	5.9	4.7	3.5	7%	−19%	−26%

The predictions for 2032* are estimated using the default output for the APC model. 1972*denotes 1971–1973; 1992* denotes 1991–1993, 2012* 2011–2013 and 2032* 2031–2033.

*OAC* oesophageal adenocarcinoma

*OSCC* oesophageal squamous cell carcinoma

### OAC trends from 1971 to 2013, and projections up to 2037

For OACs the absolute increase in new cases per year between 1972 and 2012 was much greater for men (increase by 3000 new cases) than for women (781 new diagnoses), but the relative increase was more similar (about 455% for men and 353% for women) (Table 1 and Fig. 1b). The increase in ASRs was greatest from 1972 to 1992: male ASRs increased from 4.8 to 12.3 per 100,000 (160%); female from 1.1 to 3.0 (168%). From 1992 to 2012, the increase in OAC cases was less (both in relative and in absolute terms) with increases in ASRs of 38% (12.3 to 17.0) for men and 24% (3.0 to 3.8) for women. Trends started levelling off earlier for women than for men, but then start to increase again towards the end of the projected period. Based on these trends the incidence rates of OACs were predicted to stay stable with a slight overall decrease by 5% for men and a slight overall increase by 6% for women until 2032.

### OSCC trends from 1971 to 2013, and projections up to 2037

OSCC rates decreased overall over the past 40 years for both men and women. Despite an initial small increase in the number of squamous cases between 1972 and 1992 in men (Table 1), the rates for OSCCs in men have been decreasing since 1972 corresponding to an increase in the elderly population. For women, the ASRs first increased (ASR 5.5 to 5.9) from 1972 to 1992 and then decreased from 1992 to 2012. The projected ASRs for OSCCs indicate that a further reduction in rates is to be seen from 2012 to 2037 (Fig. 1c and Table 1): male ASR projected decrease from 4.9 to 4.6 per 100,000 (−7%); female from 4.7 to 3.5 per 100,000 (−26%).

### OAC and OSCC trends and projections by age group

**Fig. 2:**
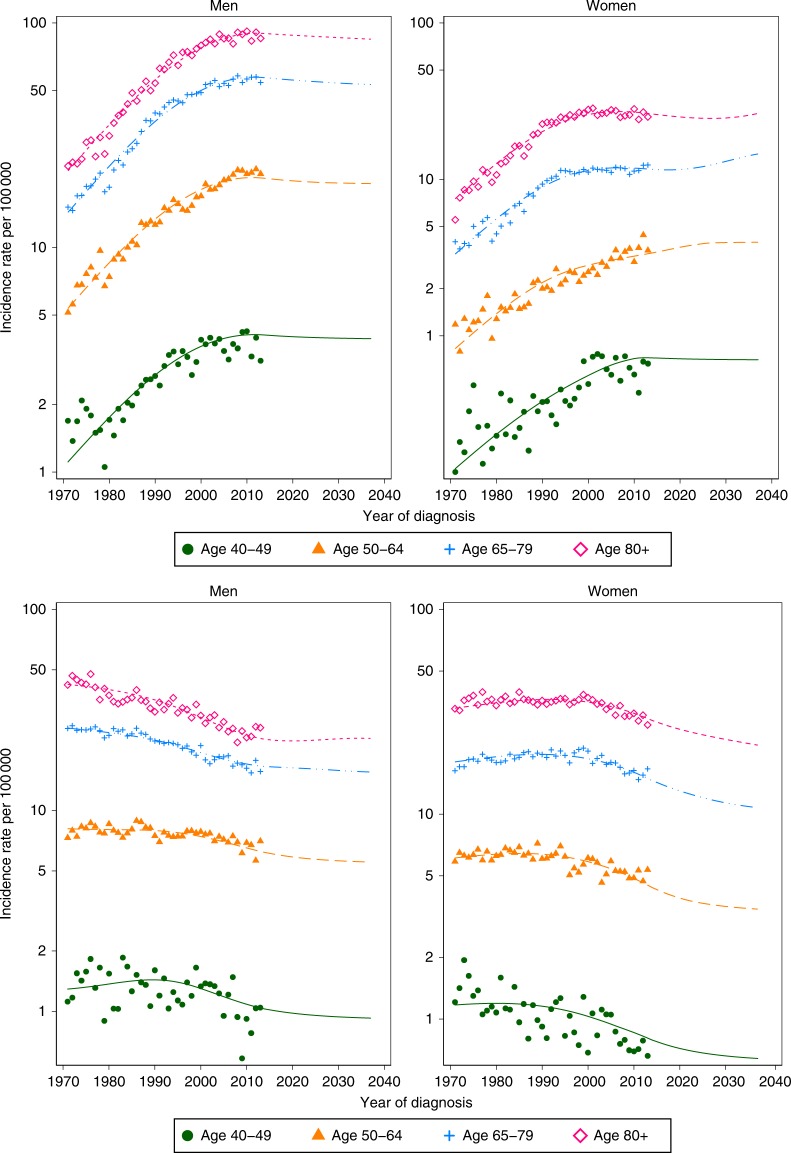
Estimated and projected incidence rates for different age ranges in men and women from 1971 to 2037 (log scale) based on an APC model. **a** OACs; **b** OSCCs; Dots represent observed + imputed rates; lines represent modelled rates using the APC model. Age groups as described in the figure legend

The increase in OAC rates from 1971 to 2013 was larger in the older age category for both genders (Fig. 2a): OAC rates for 40–49 year old men only increased from 1.7 to 3.1, whereas rates for 80 + year olds increased from 23.0 to 84.1 per 100,000. Overall, the rates for women were much lower, but also increased by age, with the largest increase for 80 + year old women (5.3 to 24.9 per 100,000). Incidence rates are predicted to level off for all age groups for both men and women.

The decrease in OSCC rates for men from 1971 to 2013 was most dramatic in the older age groups (42.3 to 25.1 for 80 + year olds and 22.9 to 14.7 for 65–79 year olds), compared to a smaller decrease for the younger age groups (7.1–6.6 for 50–64 year olds and 1.1–1.0 for 40–49 year olds; Fig. 2b). Rates are predicted to further decrease slightly for all age groups apart from the 65–79 years olds for whom rates are predicted to increase slightly to 15.2.

### Model comparisons using different age/period/cohort models

**Fig. 3:**
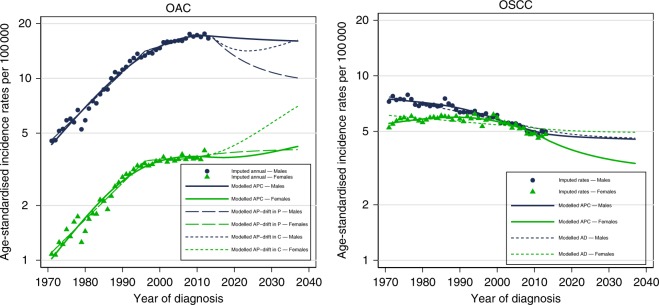
Estimated and projected incidence rates using different models for OACs and OSCCs for men and women in England from 1971 to 2037 (log scale). **a** OAC: Projections using AP or APC models. Small dashed line, estimated rates based on an AP model with the linear increase (drift) as the cohort effect; large dashed line, AP model with the linear increase (drift) as the period effect; solid line, APC model. **b** OSCC: Solid line, projections using the APC model; Small dashed line, estimated rates based on an age drift (AD)

To better understand how age, period (year of diagnosis) and cohort (year of birth) effects impact incidence rates and therefore predicted future rates further analyses using less complex models were carried out. More details on the comparison of the APC and simpler nested models can be found in the Supplementary Appendix Section 5. For OACs, compared to the standard APC model, the AP model was found to describe the data equally well for both men and women (Table S2). We, therefore, chose to compare two different simpler AP models with the more complex APC model. We firstly considered that the AP model would best describe the natural history of OACs if the linear increase is captured by a cohort effect (mostly obesity and GERD; Fig. 3a, dotted line). However, to capture the uncertainty in the AP model, and to better understand the influence of the period effect, we show an alternative solution describing the linear term as a period effect (Fig. 3a, dashed line). For both we assume that the period effect is due to the introduction of PPIs causing a reduction in cancer incidence, so we set the period effect to 0 until 1997, about 10 years after their introduction (Figure S5a). All models fit the observed data well (Fig. 3a). However, the projections for the OACs differed more dramatically depending on whether the two AP models with different options for the linear increase (drift) or the APC model was used (Fig. 3a). With the AP model describing the linear increase entirely as a cohort effect (dotted line) the rates for men are predicted to first decrease up to the early 2020s before they increase again nearing the 2012 rate. Rates for women on the other hand are predicted to increase again up to 2037. For the AP model describing the linear term as a period effect (dashed line) a decrease in rates was observed for men, whereas the rates for women stayed flat. Therefore, depending on the model, overall ASRs for men are predicted to decrease between 5 and 38% (Table S3). For women, ASRs are going to increase ranging by between 6% to 167%.

For OSCCs, model fit comparisons (Table S2) showed that a simpler model only including the age effect and a linear component (age–drift (AD) model) might be sufficient to describe the data, however, when this model is compared with the fit of the APC model, it can be seen that the trends estimated using the AD model (dashed line) did not fit the data as well as using the APC model (solid line) (Fig. 3b).

## Discussion

### Main findings

Using multiple imputations to predict missing morphology allowed us to more accurately estimate trends for OAC and OSSC rates based on over 220,000 patients diagnosed over a 42-year period in England. We found a more than 200% increase in OACs rates for both genders, with a slightly higher (relative) increase for men over this time period. The increase in OAC rates over time was larger for older age groups in both genders. Predictions of OAC rates up to 2037 differed depending on the model used: The AP model with the linear increase as a cohort effect predicted an initial dip in incidence followed by an increase for men and just a further increase in incidence rates for women. The APC model on the other hand predicted incidence rates levelling off. OSCC rates started off higher than OAC rates for both genders in 1972, then decreased slightly, and are now significantly lower than OAC rates for men. These are predicted to further decrease.

### Meaning of the study

The dramatic increase in OAC rates will most likely have been caused by a combination of the main risk factors GORD, BO and obesity. High prevalence of obesity has been linked to high incidence of OAC ^27^ and both overweight and obesity rates in England have been increasing steadily.^28^ Furthermore, the incidence of GORD has also risen worldwide in the last 40 years,^29^ and the prevalence in the Western world is now estimated to range between 10% and 20%.^30^ This could have further contributed towards the high increase in OAC incidence.

If both period and cohort effects are combined in a standard APC model without any assumptions, the predicted incidence rates levelled off for both men and women and rates are projected to stay stable. This projection depends on making “default” assumptions to identify the period and cohort effects. However, to better understand how the different risk increasing and risk reducing factors impact incidence trends we looked at less complex models (AP) in more details as they offered a good fit to the data. Firstly, we assumed that levels of obesity, GORD and BO would vary by birth cohort, i.e. increased for later cohorts, and, therefore, the contribution of these factors on the increase of OAC incidence would be in the form of a cohort effect. Secondly, we assume that the period effect was due to an increase in the use of PPIs, which have been shown to reduce the risk of OAC by 71% in a meta-analysis.^31^ We, therefore, expect the period effect only to have started to occur from the late 1990s, about 10 years after the introduction of PPIs. In 1997 3 to 4% of adults in England were estimated to use PPIs regularly, which increased to 14% in the 2010s.^32^ For an alternative model to the standard APC model we hypothesised that until the late 1990s only risks factors acting as cohort effects (mainly obesity and GORD) influenced OAC rates, which were then counteracted by risk reducing PPIs from the late 1990s. This is supported by the fact that an AC model provided the best fit for this data up to and including 1996 (not shown). Hence, we included a period effect from 1997 onwards in the AP model, which results in a dip in the projected rate for men between the early 2010s and 2020s, after which the rate increases again. This would be due to the period effect levelling off due to a saturation of the number of people taking PPIs and the cohort effect still increasing over time as environmental risk factors are still on the increase. For women on the other hand, the OACs incidence rate keeps increasing, despite a projected levelling off of the period effect. If the linear increase in rates in the AP model is only described as a period effect the projected rates drop without increasing again. This would support the hypothesis that the period effect causes a drop in the rates, whereas the cohort effect results in a further increase in men.

OSCC has been strongly linked with smoking and alcohol.^7,33–35^ The proportion of both men and women who smoke in England has decreased from over 42% for men and 36% for women in 1980 to 20% and 19%, respectively in 2010.^36^ This reduction could be largely responsible for the reduction in OSCC incidence observed here. The effect of the reduction in smoking is most likely a combination of a cohort and period effect, with fewer people starting to smoke (i.e. cohort effect) and more individuals quitting (i.e. period effect).

### Findings in relation to other studies

The majority of studies investigating OACs and OSCCs trends in Europe and North America are summarised in Table S4 in Section 6 of the Supplementary Appendix.^4–6,37–47^ We are only focusing on the Western world here as both OAC and OSCC trends are very different in Asia and other low and middle income countries.

These studies report dramatic increases in OAC rates for both genders for the majority of countries, however, a levelling off was observed in some of the studies. Two international studies by Edgren et al. and Arnold et al. found a consistently dramatic increase in incidence rates of OACs for countries from Australia, North America, Asia and Europe.^4,42^ Edgren and colleagues observed a continuous increase for men and women in England during their observation period up to 2008, whereas Arnold and colleagues found a first a rapid increase up until the mid-1990s followed by a slightly slower increase until the late 2000s, more in line with our observations. They furthermore observed a slowing increase for Australia from 1990 and slight decrease in incidence for Denmark from about 2000. Furthermore, Xie and colleagues reported a slowing down of the increase in OAC in Sweden after 2005.^47^

Using an APC model Arnold et al. predicted the OAC rates to further level off until 2030,^42^ similar to our predictions using the standard APC model. In the United States (US) three different mathematical models were used to project the OAC incidence up to 2030 using observed rates from 1975 to 2010.^5^ All three projections showed that the US rates will continue to increase for both genders until 2030, however, two of the models projected the rates to slow down in males in the late 2000s. Edgren et al. also observed that the addition of the drift (linear component) to age improved the model fit significantly for all cancer registry data including England. In addition, first adding the period effect and then both period and cohort effects further significantly improved their model fit for England.

The majority of studies reported either an initial increase in OSCC rates followed by decrease or only a decrease in OSCC rates. This is in line with our findings for England of decreasing incidence trends for men for the entire time period and an initial small increase for women followed by a decrease from early 1990s. Steevens *et al*. and Arnold *et al*. on the other hand reported an increase in OSCC rates in UK from 1982 to 1997 ^41^ and 1990 to 2000 ^42^ respectively. However, they did not include any cancers with missing morphology records. We observed that the proportion of cancers with missing morphology was higher for earlier time periods (p < 0.001), see Figure S2. Without including these cancers we also observed an increase in OSCC rates over this time period. However, once missing morphology was imputed we observed a decrease for men and a small increase for women followed by a decrease from the early 1990s onwards. Arnold et al. predicted a slight decrease in rates for men and women. Our predictions are very similar for men, however, for women our model predicts a fairly steep decrease in rates. This could be due to the fact that they are only using data from 1988 to 2007, whereas our predictions are based on records from 1971 to 2013, and we included all cancers with missing morphology.

Walther and colleagues observed that for women both the AP and AC models fitted the data well, but for men only the AP model provided a good fit.^46^ As data were only available up to 1997 for this analysis, they might have not have been able to fully see the impact of a reduction of smoking as occurred in the UK.

### Strengths and limitations

Our study has several limitations typical of studies using cancer registry data. Firstly, site and morphology records for the earlier years were not as extensive and reliable as in recent years. The proportion of oesophageal cancers with missing morphology was as high as 50% in the 1970s. However, by using all available variables to impute missing morphology, we were able to include these cancers. This resulted in more accurate cancer trends than previously reported. Secondly, the proportion of oesophageal cancer with missing morphology only started to dramatically decrease in the 1990s reaching below 20% by 2000, which then drops to less than 10% by 2013. Given that large proportions of morphology had to be imputed for earlier years, the confidence in our predictions decreases the further away they get from 2013. Thirdly, over the time period studied, the way oesophageal and stomach cancers have been recorded has changed. With the increase in interest in OAC as BO became better known in the 1980s, it is possible that cancers of the oesophagogastric junction (OGJ) have progressively become more likely to be recorded as oesophageal as opposed to cardia stomach. Furthermore, changes to the classification of cancers in the OGJ in newer editions of the American Joint Committee on Cancer (AJCC) TNM classification system could have impacted OAC trends. Whereas, the 6^th^ edition of the AJCC Cancer Staging Manual (2002) specifies that tumours arising within the OGJ and gastric cardia that only have minimal involvement (less than 2 cm) in the oesophagus should be considered primary gastric cancers,^48^ the 7^th^ edition (2009) specifies that cancers located in the OGJ or cardia that extend into the OGJ are to be staged as oesophageal cancers.^49^ However, when comparing incidence rates for OACs and cancers of the cardia, these changes in classifications of tumour locations between different editions of the AJCC staging manuals was not reflected in changes in cancer rates (data not shown). It would have been beyond the scope of this study to further assess the extent to which misclassification or changes in classification of cardia stomach cancer could lead to the observed trends in OAC. Thirdly, future predictions of cancer rates may depend on future changes to risk factors, however, data on changes in prevalence of these risk factors for the time period analysed was not available.

## Conclusion

Including cancer registry data for England up to 2013 and using multiple imputation to allow inclusion of cancers with missing morphology enabled us to include cancers not included in any previous study, therefore obtaining a more accurate model of oesophageal cancer trends until 2037. We provide evidence that the dramatic increase in OAC observed in England since the 1970s has slowed and is likely to level off. We also observed a more significant decrease in OSCC rates for both genders. We also present several models taking different effects of risk increasing and reducing factors into account to allow for the limitations of the APC model in predicting future trends. The projections of cancer incidence modelled for OSCCs and OACs presented here will provide an accurate and up to date baseline for future planning of cancer resources for both cancer subtypes.

## Electronic supplementary material

Supplementary Appendix

## Availability of data

The data set used here can be obtained from the PHE Office for Data Release. The authors can provide data specifications for any data requests to the Office of Data Release.
