# Supplementary material for: Trends and projections in adenocarcinoma and squamous cell carcinoma of the oesophagus in England from 1971 to 2037
Source: Br J Cancer. 2018 Mar 22;118(10):1391–8. doi: 10.1038/s41416-018-0047-4 (PMC5959941; doi:10.1038/s41416-018-0047-4)
Supplement: Supplementary file 1 — Supplementary Appendix [file 41416_2018_47_MOESM1_ESM.docx]

**Supplementary Appendix**

**Cited in Methods:**

**Section 1:**

**Missing morphology and imputation**

In addition to cancers with missing morphology codes, cancer with ICD-O codes 8000-8005 (neoplasm, malignant tumour) and 8010-8015 (carcinoma not otherwise specified) were also considered to have ‘missing’ morphology, as it is not possible to categorise them. To estimate missing data it was assumed that morphology was missing at random conditional of various factors. A multinomial logistic regression model was fitted with outcome morphology type (OAC, OSCC or other) using age group, gender, year of diagnosis, cancer sub-site, basis of diagnosis as explanatory variables. The predictors included in the imputation model were selected based on their association with missingness of morphology type, for example year was negatively associated with missing information while missing information increases with age. Moreover, an interaction term between decade of diagnosis and age in 15-year group (e.g. 40-54, 55-69) was also included to reflect different patterns of reduction in missing data across age groups.

Using the results of this model, the probability of either morphology category was then predicted for each individual cancer with missing information. This was repeated 50 times (i.e. multiple imputation). All further analyses were conducted separately on each imputed dataset and results were, subsequently, combined using Rubin’s rules (White *et al*, 2011). The standard deviation was calculated as the square root of the sum of the variance within and between each imputation (Marshall *et al*, 2009). To determine the accuracy of the MI model at estimating morphology, we compared the observed versus estimated proportion of adenocarcinomas across strata (i.e. by sex and age) together with the difference (observed – estimated). The interquartile range (IQR) across imputations was calculated as a measure of variability across strata.

**Section 2:**

**Age Period Cohort Models**

The cubic splines for period and cohort were reduced to 2 knots each. A regular, linear increase or decrease is termed ‘drift’ and could be attributable to linear period, linear cohort or both. When making future predictions Møller *et al.* believed it valuable to dampen the drift based on the belief that past trends will not continue forever (Moller *et al*, 2003). We therefore applied a dampening factor to the drift when extrapolating to allow current trends to be attenuated over time. We reduced the drift by 8% each year for the period after our observations, so that the drift after 8 years was approximately half of the effect in the observed period.

**Different APC modeling approaches**

We compared a number of models (i.e., age, age-drift, age-period (AP), age-cohort (AC) and, age-period-cohort (APC) models), to identify the one that best fit our data using the McFadden’s pseudo R-squared as the measure of goodness of fit rather than significant testing. For APC models, alternative solutions for the period and cohort effects are presented in each graph. All models are centred so that the relative risk is 1 in the year 1990 and cohort 1925 (i.e. age 65), which represents the anchoring point. For the AP model for OACs, the relative risk as a function of period was set to 1 up to and including 1996. In one of the AP models, we included the linear term (i.e. drift) for the period effect together with the period non-linear components (captured by the splines). We applied geometric damping to this linear term so that after 8 years the drift is halved. In the second model, we included the linear term for the cohort together with the period non-linear components. We applied as a restriction that future cohorts should have the same effect as the last observed cohort. By specifying the drift as either cohort or period the damping applied to the model differs resulting in different overall projections.

**Cited in Results**

**Section 3:**

**Missing morphology and imputation**

The percentage with unknown morphology decreased from 46% in 1971-75 and to below 10% in 2011-13 (Figure S1). Figure S2 shows the number of OACs and OSCCs including cancers with imputed missing morphology averaged across 50 imputations, with the error bars showing only small within and between imputation variability. Figure S3 shows the morphology distribution of all oesophageal cancers diagnosed between 1971 and 2013 without and with cancers with imputed morphology. The slight decrease in the proportion of OAC amongst all cancers from 0.54 to 0.52 and slightly increase in the proportion of OSCCs from 0.43 to 0.45 could be explained by the fact that the proportion of cancers with missing morphology was highest in the 1970s and 80s (Figure S1) when the incidence of OACs was still lower. The IQR as a measure of variability (Table S1) indicates little variation across MIs for the different strata. Furthermore, the imputed proportion of adenocarcinomas reflected the one in the observed (complete) data, which indicates high accuracy of the MI model at estimating morphology.

Comparing ASRs of OACs and OSCCs based on recorded morphologies only (Figure S1) with ASRs of cancers combining both recorded and imputed morphologies (Figure 1), it can be seen that rates for both OACs and OSCCs were shifted upwards for earlier years. This resulted in an overall larger decrease of OSCC rates as, for example ASRs were about twice as high in the 1970s for men compared to raw data. Including imputed OACs cases resulted in a smaller overall increase in ASRs for men with rates starting about twice as high in the 1970s when imputed cases were included. A similar, but not as dramatic effect was observed for women**.**

**Figure S1**. Incidence rates for OACs and OSCCs for men and women in England from 1971 to 2013 (log scale). Annual incidence rates of cancers with recorded morphology only (left y-axis) and proportion of cancers with missing morphology (right y-axis) for men and women. OAC, adenocarcinoma; OSCC, squamous cell carcinoma; ASR, age standardised incidence rates; ESP, European standard population.


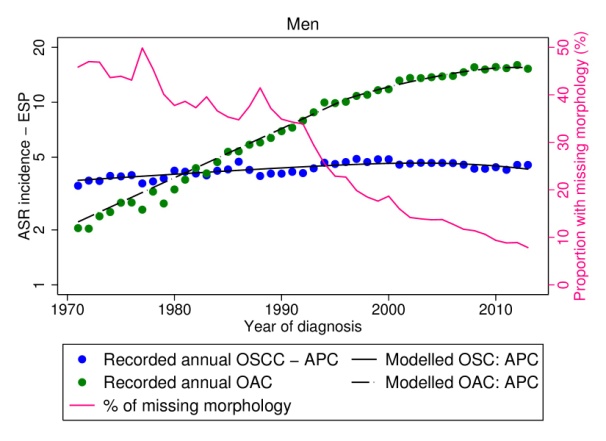


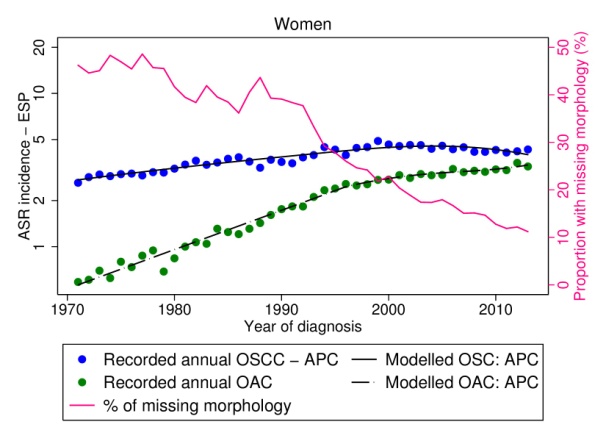


**Figure S2.** Mean number of newly diagnosed OACs and OSCCs based on oesophageal cancers with known morphology in addition to cancers with predicted morphology based on multiple imputation (50 imputed datasets) for cancers with missing morphology data by year of diagnosis. Error bars: standard error term consisting of the square root of the sum of within and between the variance. (a) OACs; (b) OSCCs.

(a)


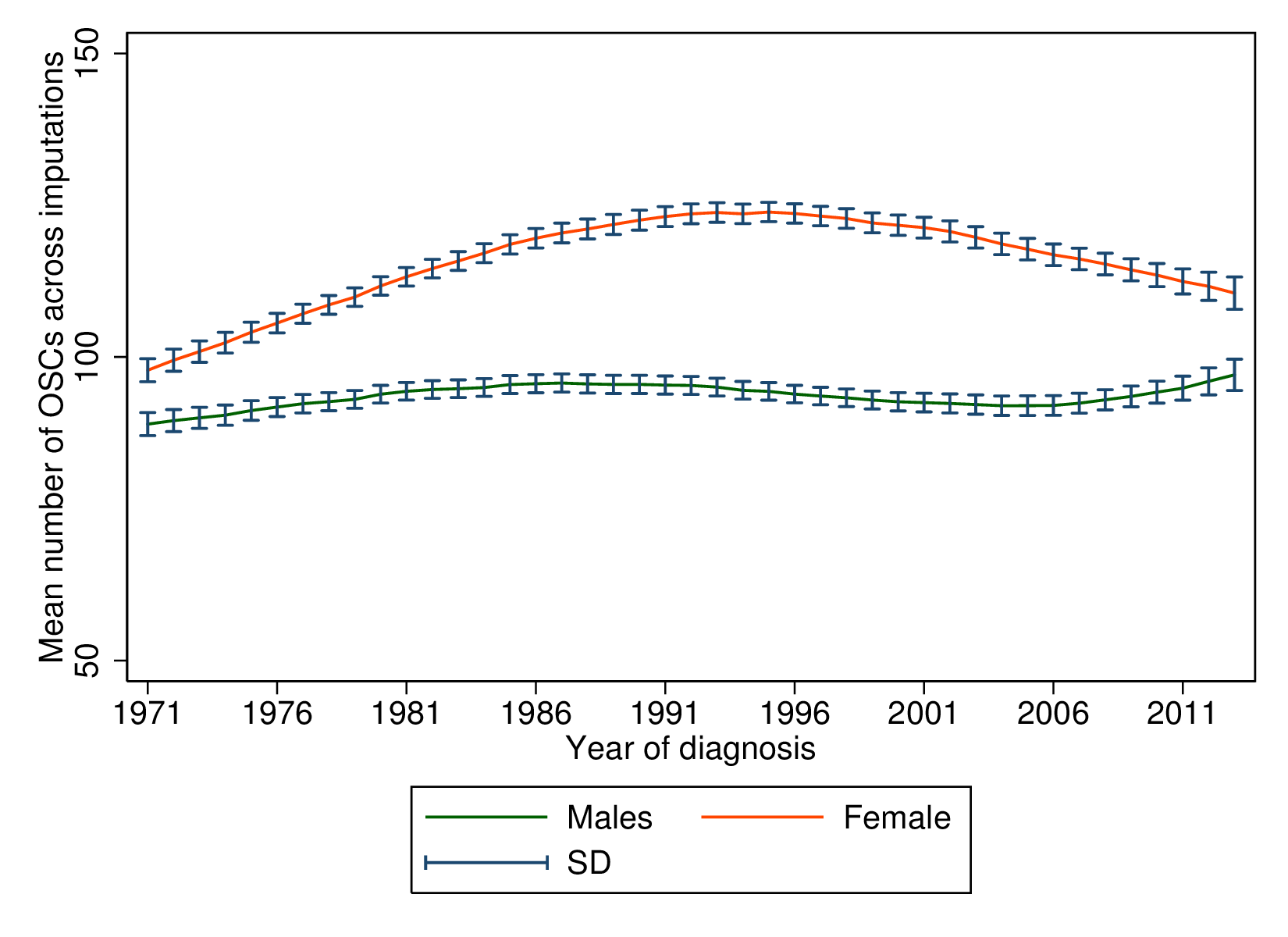


(b)


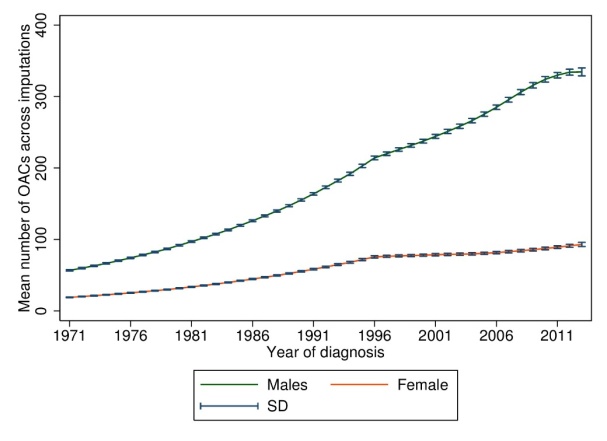


**Figure S3:** Distribution of all OACs, OSCCs and other cancers newly diagnosed between 1971 and 2013 amongst cancers with known morphology compared to amongst cancers with know and imputed morphology. (a) cancers with known morphology only; (b) cancers with known and imputed morphology.

(a)


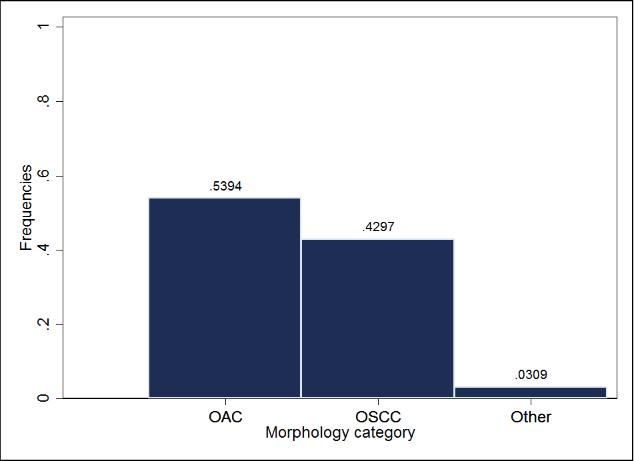


(b)

**
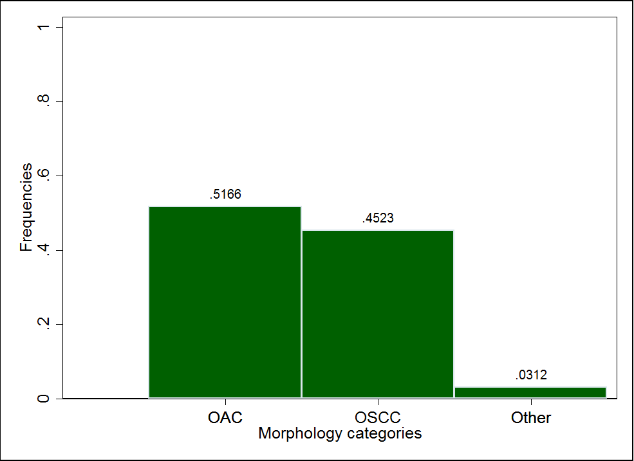
**

| Table S1. Variability across imputed morphology for oesophageal adenocarcinomas. Difference = observed-estimated | | | | | |
| --- | --- | --- | --- | --- | --- |
| **Stratum** | | **Percentage of OACs (%)** | | |  |
| **Sex** | **Age** | **Imputed** | **Observed** | **Difference** | **IQR** |
| **Male** | **<39** | 71.8 | 72.7 | 0.77 | 0.016 |
| **Male** | **40-49** | 67.4 | 68.6 | 1.18 | 0.008 |
| **Male** | **50-64** | 64.1 | 65.4 | 1.26 | 0.004 |
| **Male** | **65-79** | 65.7 | 65.6 | 0.10 | 0.003 |
| **Male** | **80+** | 71.2 | 68.8 | 2.35 | 0.006 |
| **Female** | **<39** | 38.3 | 36.6 | 1.70 | 0.046 |
| **Female** | **40-49** | 32.8 | 29.3 | 3.53 | 0.012 |
| **Female** | **50-64** | 29.9 | 26.9 | 3.01 | 0.007 |
| **Female** | **65-79** | 31.8 | 32.1 | 0.24 | 0.003 |
| **Female** | **80+** | 37.8 | 40.0 | 2.20 | 0.005 |
| IQR. Interquartile range | | | | | |

**Section 4:**

**Trends and predictions of recorded data without MI**

Comparisons of trends and projections of the APC model with and without imputed data can be seen in Figure S4. For OACs both curves have similar shapes, however, incidence rates without MI start at about half of the incidence with MI. The increase in rates without MI is therefore steeper between 1970s and early 2000s for both men and women, while predicted rates are also lower than when imputed cancers are included. Furthermore, for OSCCs, past trends without MI indicate an increase rather than a decrease in incidence (as reported in the literature) compared to only a decrease in rates observed with MI. This further results in a projected steeper decrease in rates without imputed cancers. Not including cancers could therefore result in an underestimate of future OAC and OSCC incidence rates.

**Figure S4:** Incidence rates for OACs and OSCCs with and without cancer with imputed morphology for men and women in England (log scale). Dots represent observed rates; lines represent modelled rates using the APC model. Purple graphs show rates for recorded cancers only, orange graphs present recorded and imputed cancer. OAC, adenocarcinoma; OSCC, squamous cell carcinoma; ASR, age standardised incidence rates; ESP, European standard population.

**
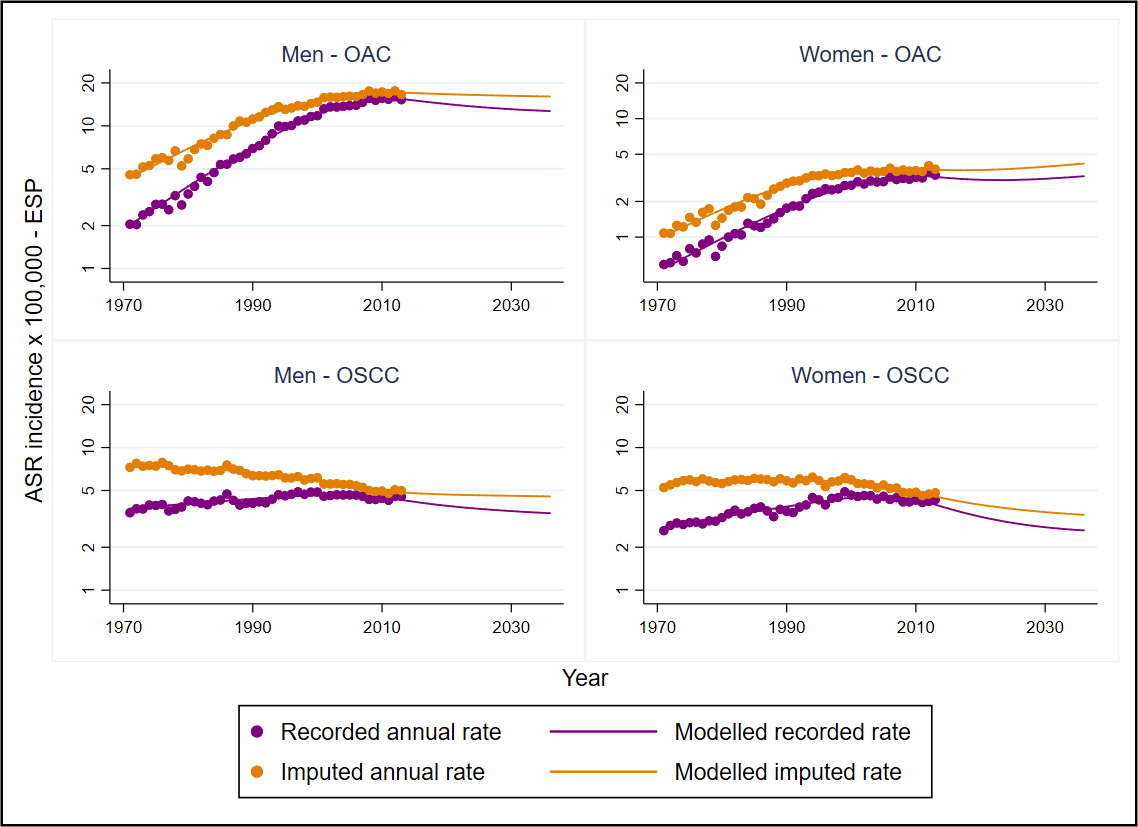
**

**Section 5:**

**Model comparison using different age / period / cohort models**

| **Table S2.** Model fit comparisons between the APC spline and simpler nested models using the exponential link function and power function. The drift was included in all models from the AD model onward. The model selected for all further analysis is highlighted in bold. | | | | | | | |
| --- | --- | --- | --- | --- | --- | --- | --- |
|  |  |  |  |  |  |  |  |
|  |  |  |  |  |  |  |  |
|  |  |  |  |  |  |  |  |
|  |  | **Male** | | | **Female** | | |
|  | **df** | **X^2^ gof** | **X^2^/df** | **Pseudo R^2^** | **X^2^ gof** | **X^2^/df** | **Pseudo R^2^** |
| **OAC** | | | | | | | |
| **Exponential function** | | | | | | | |
| A | 465 | 11877.7 | 25.5 | 0.9285 | 3543.9 | 7.6 | 0.9196 |
| AD | 464 | 1746.5 | 3.8 | 0.9782 | 1030.3 | 2.2 | 0.9553 |
| **AP** | **462** | **957.5** | **2.1** | **0.9818** | **631.1** | **1.4** | **0.9607** |
| AC | 462 | 1508.1 | 3.3 | 0.9792 | 848.2 | 1.8 | 0.9577 |
| **APC** | **460** | **927.5** | **2** | **0.9819** | **579.9** | **1.3** | **0.9615** |
|  | | | | | | | |
| **Power function** | | | | | | | |
| A | 465 | 11756.5 | 25.3 | 0.9289 | 3534.6 | 7.6 | 0.9197 |
| AD | 464 | 2314.6 | 5 | 0.9759 | 1138.8 | 2.5 | 0.9546 |
| AP | 462 | 2012.3 | 4.4 | 0.9783 | 974.7 | 2.1 | 0.9581 |
| AC | 462 | 1229.7 | 2.7 | 0.9804 | 795.7 | 1.7 | 0.9586 |
| APC | 460 | 1057.9 | 2.3 | 0.9813 | 714 | 1.6 | 0.9601 |
| **OSCC** | | | | | | | |
| **Exponential function** | | | | | | | |
| A | 465 | 1478.1 | 3.2 | 0.9616 | 972.8 | 2.1 | 0.9713 |
| AD | 464 | 800.8 | 1.7 | 0.9676 | 835.7 | 1.8 | 0.9723 |
| AP | 462 | 761.5 | 1.6 | 0.9679 | 689.1 | 1.5 | 0.9735 |
| AC | 462 | 761.8 | 1.6 | 0.9679 | 729.9 | 1.6 | 0.9731 |
| **APC** | **460** | **662.9** | **1.4** | **0.9688** | **642.5** | **1.4** | **0.9737** |
| **Power function** | | | | | | | |
| A | 465 | 1418.1 | 3.1 | 0.962 | 924.6 | 2 | 0.9716 |
| AD | 464 | 839.8 | 1.8 | 0.9671 | 760.3 | 1.6 | 0.9728 |
| AP | 462 | 801.7 | 1.7 | 0.9676 | 636.1 | 1.4 | 0.9738 |
| AC | 462 | 731.3 | 1.6 | 0.9681 | 694.6 | 1.5 | 0.9733 |
| APC | 460 | 614.7 | 1.3 | 0.9692 | 610.7 | 1.3 | 0.9739 |

df, degrees of freedom; gof, goodness of fit; A, age; P, period; C, cohort; D, drift.

Model fit comparisons APC and simpler models are shown in Table S2. The model fit indices (e.g. pseudo R-squared) indicate the APC model with 6 knots for the age effect and 2 knots for the period and cohort effects offer a good fit to the data. As using the default number of knots (6 for age, 5 for period and 3 for cohort) did not further improve the model fit (not shown), we felt that 2 knots were sufficient to smooth the data.

Owing to the identifiability issue inherent in the APC model, we are showing alternative “solutions” based on rotations of the period and cohort effects around the anchoring point (the constraint). In particular, we were interested in exploring how the period and cohort effects depend on how the drift is allocated, as can be seen for these individual effects in Figure S5a. However, the differences in predicted trends is small for OACs (Figure S6a), so the default APC and two AP models were used for the ASR predictions (Tables 1 and S2).

For SCCs, alternative solutions rotating the terms of the APC model around the same anchoring points (Figures S5b) were similarly estimated. Age group specific projections varied depending on the model used: For OSCCs, the different rotations resulted in nearly identical projections for women and only slightly differing projections for men with the biggest differences observed in older men (Figure 6d). As predicted rates don’t vary as a function of the rotation (Figure S5b) the default APC was used for the ASR predictions.

These rotations do not affect the projections per se but are used to show how period and cohort effects can vary in interpretation depending on the criteria applied to the model, specifically rotation around the anchoring point.

**Figure S5:** Estimates of the age effect as absolute risk and period and cohort effects as relative risk for oesophageal cancer incidence in England: (a) AP and APC models for adenocarcinomas; (a) APC model for squamous cell carcinomas. For both APC models: green line, default; orange and blue lines, rotations around relative risk of 1 in 1990 and for 1925 cohort for period and cohort effects respectively; males (top panels), females (bottom panels). For the AP model for ACs: the period effect was fixed to 1 up to 1996 (red crosses), and only fitted to the data from 1997;


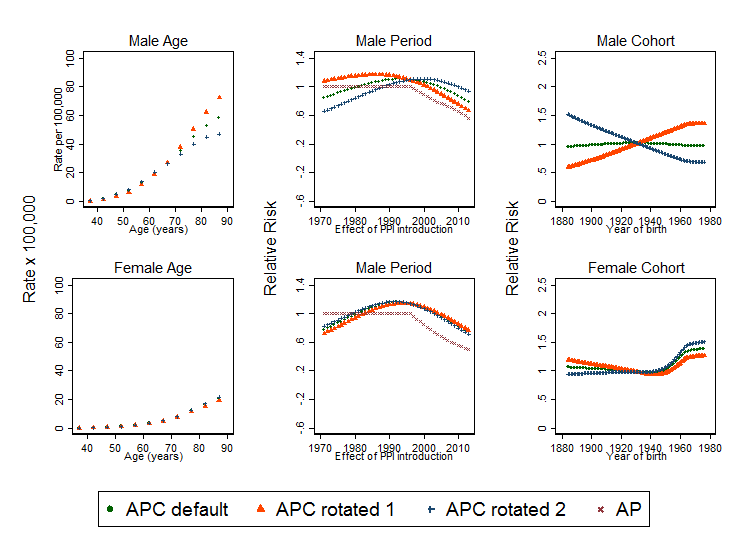
(a)


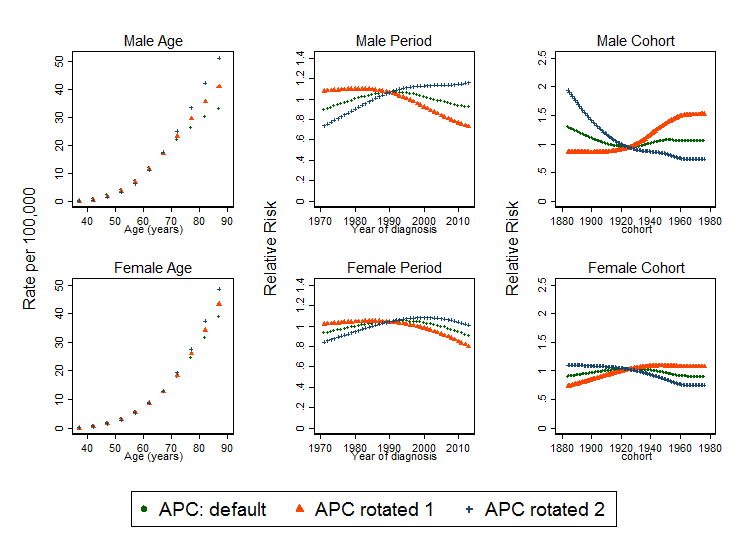
(b)

**Figure S6**. Estimated and projected incidence rates for different age ranges in men and women from 1971 to 2037 (log scale). (a) OACs and (b) OSCCs: Projections using the APC model showing alternative solutions obtained by rotating the output. Solid lines, based on the default model; finely dotted lines based on a model where constant subtracted from period but added to cohort; dashed line: opposite model where constant added to period and subtracted from cohort.


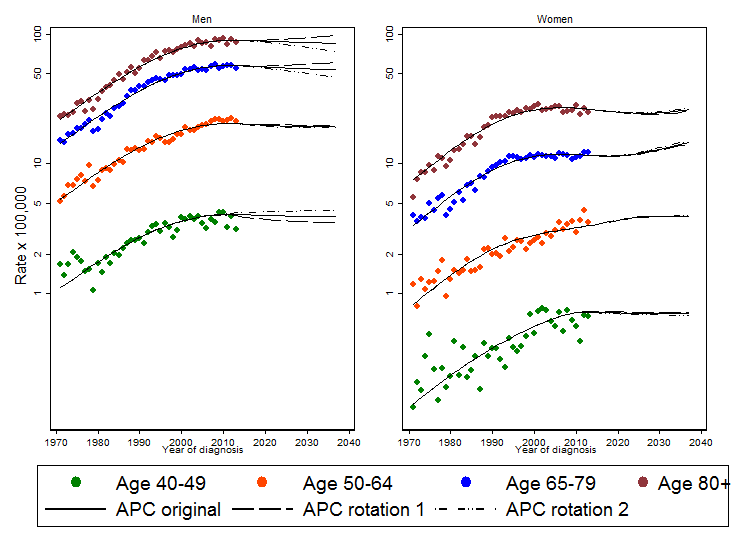
(a)


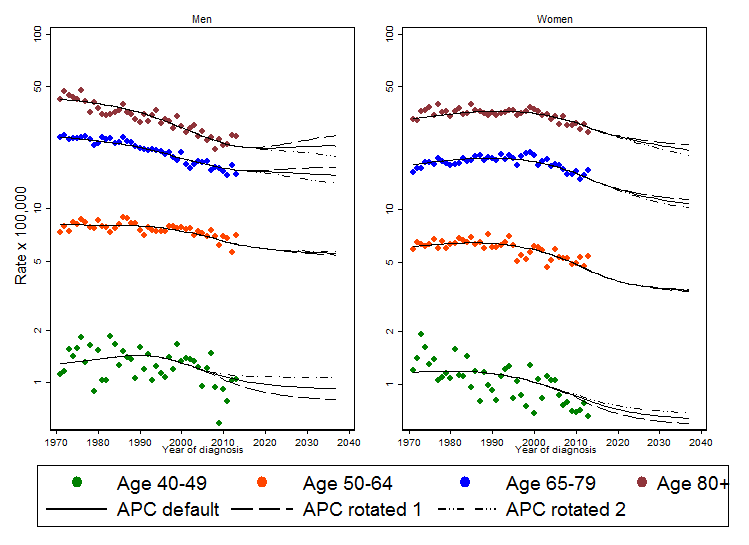
(b)

| **Table S3:** Predicted age-standardised incidence rates and numbers of oesophageal adenocarcinomas in men and women based on the APC or AP model with the drift either in the period or cohort effect. 2032*denotes the moving average for 2031-2033. | | | | | | | | | | | | |
| --- | --- | --- | --- | --- | --- | --- | --- | --- | --- | --- | --- | --- |
|  |  |  |  |  |  |  |  |  |  |  |  |  |
|  | **APC model** | | | | **AP model with drift in period effect** | | | | **AP model with drift in cohort effect** | | | |
|  | **Average number of cases** | | **ASP (per 100,000)** | | **Average number of cases** | | **ASP (per 100,000)** | | **Average number of cases** | | **ASP (per 100,000)** | |
|  | **Cases in 2032*** | **Total change 2012*-2032*** | **ASP** | **Total change 2012*-2032*** | **Cases in 2032*** | **Total change 2012*-2032*** | **ASP** | **Total change 2012*-2032*** | **Cases in 2032*** | **Total change 2012*-2032*** | **ASP** | **Total change 2012*-2032*** |
| **Sex** | | | | | | | | | | | | |
| **Male** | 5191 | 42% | 16.2 | -5% | 3370 | -8% | 10.5 | -38% | 4935 | 35% | 15.2 | -11% |
| **Female** | 1531 | 53% | 4 | 6% | 1596 | 59% | 4 | 7% | 2448 | 144% | 10.8 | 187% |
| OAC, adenocarcinoma; OSCC, squamous cell carcinomas. | | | | |  |  |  |  |  |  |  |  |

**Section 6:**

**Discussion**

| **Table S4.**  Summary on major studies of trends in incidence of oesophageal adenocarcinomas or squamous cell carcinomas | | | | | | |
| --- | --- | --- | --- | --- | --- | --- |
|  |  |  |  |  |  |  |
| **Study** | **Time period** | **Country** | **Histological subtype** | **Missing morphology** | **Methodology** | **Observed trends** |
| Xie et al. (Xie *et al*, 2017) | 1970 - 2014 | Sweden | OAC | Not reported | Joinpoint Regression Analysis | Slow increase until 1994 followed by more rapid increase from 1994-2000 followed by slowing of increase for men. Slow increase for women. |
| Edgren et al. (Edgren *et al*, 2013) | 1971 - 2009 | Global | OAC | Not reported | APC models; simpler models | Consistent dramatic increase in incidence starting between 1960 and 1990. |
| Vizcaino et al. (Vizcaino *et al*, 2002) | 1973 - 1994 | International | OAC | Countries with >25% missing morphology excluded; For countries included cases with missing morphology ranged from 5 to 23% | APC model; simpler models | Significant increases for both genders for most countries apart from the Netherlands; In France an increased incidence was observed only for men and in Switzerland only for women. |
| Kroep et al. (Kroep *et al*, 2014) | 1974-2010 | Netherlands/ Spain / USA (SEER) | OAC | Not reported | Joinpoint Regression Analysis | Incidence increased in all three countries with highest increase in the Netherlands, then USA, lastly Spain. |
| Hur et al. (Hur *et al*, 2013) | 1975 - 2009 | USA (SEER) | OAC | Not reported | Joinpoint Regression Analysis | Incidence rates continue to increase with slowing of increase occurring around 1997. |
| Kong et al. (Kong *et al*, 2014) | 1975 - 2009 + projected to 2030 | USA (SEER) | OAC | Not reported | Three different APC models (CISNET) | All models predict that incidence will continue to increase with plateauing trend for recent cohorts. |
| Thrift and Whiteman (Thrift & Whiteman, 2012) | 1984 - 2008 | Australia / USA / Sweden | OAC | Not reported | Joinpoint and APC analysis | Rapid increase between 1984 and 1994 in Australia and 1998 in the US followed by slower increase until 2008. In Sweden incidence was stable until 1993, increased between 1993 and 2001 and then remained stable. |
| Otterstatter et al. (Otterstatter *et al*, 2012) | 1986 - 2006 + projections to 2026 | Canada | OAC | Not reported | APC model (Nordpred) | Incidence rates roughly doubled for both men and women from 1986 to 2006, predicted slowing of increase occurring for men from late 2010s. |
| Arnold et al. (Arnold *et al*, 2017) | 1988 - 2007 + projections to 2030 | International | OAC | Not reported | APC model (Nordpred) | Observed rapid increase in all countries. Rates predicted to further increase with a levelling off in some countries. |
| Arnold et al. (Arnold *et al*, 2017) | 1988 - 2007 + projections to 2030 | UK | OAC | Not reported | APC model (Nordpred) | Significant increase observed and predicted up to about 2010 followed by slowing of increase for men and women. |
| Noone et al. (Noone *et al*, 2017) | 1992-2013 | USA (SEER) | OAC | Not reported | Joinpoint Regression Analysis | Increased incidence for men and stable incidence for women. |
| Trivers et al. (Trivers *et al*, 2008) | 1998 - 2003 | USA (SEER) | OAC | 6% of all OCs unspecified, not included | Age adjusted incidence rates | Increased incidence during analysed time period. |
| Edgren et al.(Edgren *et al*, 2013) | 1971 - 2009 | England | OAC | Not reported | APC models; simpler models | Continuous increase in incidence for men; lower increase for women. |
| Dubecz et al. (Dubecz *et al*, 2014) | 1973 - 2009 | USA (SEER) | OAC + AC of GC | Not reported | Joinpoint Regression Analysis | Dramatic increase in incidence of both OAC and AC of the cardia; increase slowed for OAC after 1997 and plateaued for AC of the gastric cardia after 1985. |
| Walther et al. (Walther *et al*, 2001) | 1970 - 1997 | Sweden | OAC and AC of GOJ | Not reported | APC models; simpler models | Increased incidence for men and women by ~20%. |
| Steevens et al. (Steevens *et al*, 2010) | 1983 - 1997 | UK | OAC and AC of GC | Not reported | Joinpoint Regression Analysis | Significant increase for men and women (roughly doubled). |
| Steevens et al. (Steevens *et al*, 2010) | 1983 - 1997 | Europe | OAC and AC of GC | Not reported | Joinpoint Regression Analysis | Increased incidence for men and women in most European regions, with largest increase in Northern Europe. |
| Walther et al.(Walther *et al*, 2001) | 1970 - 1997 | Sweden | OSCC | Not reported | APC models; simpler models | Initial increase followed by decrease for men; overall decrease for women. |
| Vizcaino et al. (Vizcaino *et al*, 2002) | 1973 - 1994 | International | OSCC | Countries with >25% missing morphology excluded; For countries included cases with missing morphology ranged from 5 to 23%. | APC model; simpler models | Incidence rates declined for men in most countries analysed apart from Denmark and the Netherlands; for women significant increase also noted for Canada, Switzerland and Australia. |
| Steevens et al. (Steevens *et al*, 2010) | 1983 - 1997 | Europe | OSCC | Not reported | Joinpoint Regression Analysis | Increased slightly for men and women in Northern Europe. Decreased for men and increased for women in Southern and Western Europe. |
| Steevens et al. (Steevens *et al*, 2010) | 1983 - 1997 | UK | OSCC | Not reported | Joinpoint Regression Analysis | Slight increase for both men and women. |
| Xie et al. (Xie *et al*, 2017) | 1970 - 2014 | Sweden | OSCC | Not reported | Joinpoint Regression Analysis | Decrease until 1974 followed by slight increase in 1974-1986 and then steady slow decrease from 1987 onwards for men; very slow overall decrease for women. |
| Thrift and Whiteman (Thrift & Whiteman, 2012) | 1984 - 2008 | Australia / USA / Sweden | OSCC | Not reported | Joinpoint and APC analysis | Significant linear declines in incidence in all three populations. |
| Arnold et al. (Arnold *et al*, 2017) | 1988 - 2007 + projections to 2030 | International | OSCC | Not reported | APC model (Nordpred) | Projected continuing decrease in most countries. |
| Arnold et al. (Arnold *et al*, 2017) | 1988 - 2007 + projections to 2030 | UK | OSCC | Not reported | APC model (Nordpred) | Initial decrease, followed by an increase and then a slight decrease for both men and women. Predicted to further decrease for men and remain the same for women. |
| Noone et al. (Noone *et al*, 2017) | 1992-2014 | USA (SEER) | OSCC | Not reported | Joinpoint Regression Analysis | Decreasing incidence for women and women. |
| Trivers et al. (Trivers *et al*, 2008) | 1999 - 2003 | USA (SEER) | OSCC | 6% of all OCs unspecified, not included | Age adjusted incidence rates | Incidence rate decreased by on average 3.6% per year. |
| Otterstatter et al. (Otterstatter *et al*, 2012) | 1986 - 2006 + projections to 2026 | Canada | OSCC | Not reported | APC model (Nordpred) | Decreased for both men and women observed, with further reduction predicted. |

OAC, adenocarcinoma; OSCC, squamous cell carcinomas; APC, Age-period-cohort; OC, oesophageal cancer; GOJ, gastro-oesophageal junction; AC, adenocarcinoma; GC, gastric cardia.

**References**

Arnold M, Laversanne M, Brown LM, Devesa SS, Bray F (2017) Predicting the Future Burden of Esophageal Cancer by Histological Subtype: International Trends in Incidence up to 2030. *The American journal of gastroenterology* **112**(8)**:** 1247-1255

Clayton D, Schifflers E (1987a) Models for temporal variation in cancer rates. I: Age-period and age-cohort models. *Stat Med* **6**(4)**:** 449-67

Clayton D, Schifflers E (1987b) Models for temporal variation in cancer rates. II: Age-period-cohort models. *Stat Med* **6**(4)**:** 469-81

Dubecz A, Solymosi N, Stadlhuber RJ, Schweigert M, Stein HJ, Peters JH (2014) Does the Incidence of Adenocarcinoma of the Esophagus and Gastric Cardia Continue to Rise in the Twenty-First Century?—a SEER Database Analysis. *Journal of Gastrointestinal Surgery* **18**(1)**:** 124-129

Edgren G, Adami H-O, Weiderpass E, Nyrén O (2013) A global assessment of the oesophageal adenocarcinoma epidemic. *Gut* **62**(10)**:** 1406-1414

Hur C, Miller M, Kong CY, Dowling EC, Nattinger KJ, Dunn M, Feuer EJ (2013) Trends in esophageal adenocarcinoma incidence and mortality. *Cancer* **119**(6)**:** 1149-1158

Kong CY, Kroep S, Curtius K, Hazelton WD, Jeon J, Meza R, Heberle CR, Miller MC, Choi SE, Lansdorp-Vogelaar I, van Ballegooijen M, Feuer EJ, Inadomi JM, Hur C, Luebeck EG (2014) Exploring the recent trend in esophageal adenocarcinoma incidence and mortality using comparative simulation modeling. *Cancer Epidemiol Biomarkers Prev* **23**(6)**:** 997-1006

Kroep S, Lansdorp-Vogelaar I, Rubenstein JH, Lemmens VEPP, van Heijningen EB, Aragones N, van Ballegooijen M, Inadomi JM (2014) Comparing Trends in Esophageal Adenocarcinoma Incidence and Lifestyle Factors Between the United States, Spain, and The Netherlands. *The American journal of gastroenterology* **109**(3)**:** 336-343

Marshall A, Altman DG, Holder RL, Royston P (2009) Combining estimates of interest in prognostic modelling studies after multiple imputation: current practice and guidelines. *BMC medical research methodology* **9:** 57

Moller B, Fekjaer H, Hakulinen T, Sigvaldason H, Storm HH, Talback M, Haldorsen T (2003) Prediction of cancer incidence in the Nordic countries: empirical comparison of different approaches. *Stat Med* **22**(17)**:** 2751-66

Noone A-M, Cronin KA, Altekruse SF, Howlader N, Lewis DR, Petkov VI, Penberthy L (2017) Cancer Incidence and Survival Trends by Subtype Using Data from the Surveillance Epidemiology and End Results Program, 1992–2013. *Cancer Epidemiology Biomarkers &amp; Prevention* **26**(4)**:** 632-641

Otterstatter MC, Brierley JD, De P, Ellison LF, MacIntyre M, Marrett LD, Semenciw R, Weir HK, for the Canadian Cancer Statistics Steering C (2012) Esophageal cancer in Canada: Trends according to morphology and anatomical location. *Canadian Journal of Gastroenterology* **26**(10)**:** 723-727

Steevens J, Botterweck AAM, Dirx MJM, van den Brandt PA, Schouten LJ (2010) Trends in incidence of oesophageal and stomach cancer subtypes in Europe. *European journal of gastroenterology & hepatology* **22**(6)**:** 669-678

Thrift AP, Whiteman DC (2012) The incidence of esophageal adenocarcinoma continues to rise: analysis of period and birth cohort effects on recent trends. *Ann Oncol* **23**(12)**:** 3155-3162

Trivers KF, Sabatino SA, Stewart SL (2008) Trends in esophageal cancer incidence by histology, United States, 1998–2003. *Int J Cancer* **123**(6)**:** 1422-1428

Vizcaino AP, Moreno V, Lambert R, Parkin DM (2002) Time trends incidence of both major histologic types of esophageal carcinomas in selected countries, 1973–1995. *Int J Cancer* **99**(6)**:** 860-868

Walther C, Zilling T, Perfekt R, Möller T (2001) Increasing prevalence of adenocarcinoma of the oesophagus and gastro-oesophageal junction: a study of the Swedish population between 1970 and 1997. *European Journal of Surgery* **167**(10)**:** 748-757

White IR, Royston P, Wood AM (2011) Multiple imputation using chained equations: Issues and guidance for practice. *Stat Med* **30**(4)**:** 377-99

Xie S-H, Mattsson F, Lagergren J (2017) Incidence trends in oesophageal cancer by histological type: An updated analysis in Sweden. *Cancer Epidemiology* **47:** 114-117
